# Co-Overexpression of *Geissoschizine Synthase* and *Catharanthine Synthase* Increases Catharanthine Biosynthesis in *Catharanthus roseus* Hairy Roots

**DOI:** 10.3390/plants15142220

**Published:** 2026-07-21

**Authors:** Xiaohua Li, Gongqiang Li, Zhiwei Hu, Jiacheng Wang, Yang Chen, Kunhong Zhu, Zichen Huang, Mingyu Wang, Jiayi Sun

**Affiliations:** College of Life and Environmental Science, Wenzhou University, Zhong-Xin Street, Wenzhou 325035, China; xiaohuali2026@163.com (X.L.); li18267782452@163.com (G.L.); zhiwei.hu@wzu.edu.cn (Z.H.); wjc18367712919@163.com (J.W.); cy2401841936@163.com (Y.C.); zkh3163458987@outlook.com (K.Z.); hzcnew@outlook.com (Z.H.); wmy6793@gmail.com (M.W.)

**Keywords:** *Catharanthus roseus*, hairy roots, catharanthine, metabolic engineering, metabolomics, *catharanthine synthase*, *geissoschizine synthase*

## Abstract

Plant-based platforms remain vital for producing anticancer vinca alkaloids. However, one major challenge in plant-based metabolic engineering is that target metabolite yields are often hindered by the presence of multiple competing pathway branches that disperse metabolic flux. To overcome these bottlenecks, we first implemented a competitive branch-point diversion strategy by co-overexpressing *geissoschizine synthase* and *catharanthine synthase* under an inducible promoter in *Catharanthus roseus* hairy roots. This targeted intervention successfully shifted metabolic partitioning, driving a peak relative increase of 67% in catharanthine accumulation at the 96 h time point, while significantly reducing competing by-products such as ajmalicine, serpentine, and tabersonine. Targeted qPCR analysis revealed a transient upregulation of core monoterpene indole alkaloid biosynthetic genes and a coordinated suppression of some competing branch genes. Despite this effective downstream diversion, overall yield enhancement was constrained by tight regulation, notably the downregulation of *BIS* transcriptional activators, which restricts the supply of upstream terpenoid precursors. Furthermore, untargeted metabolomic profiling highlighted a widespread metabolic shifts featuring the significant enrichment of phenylalanine-derived secondary pathways. Ultimately, this study demonstrates the efficacy of competitive branch-point diversion for target alkaloid enrichment, while highlighting the complex regulatory dynamics and broad metabolic alterations within the *C. roseus* network.

## 1. Introduction

*Catharanthus roseus* has long served as a model organism for investigating plant secondary metabolites of high clinical value [1]. The vinca alkaloids, which are biosynthesized exclusively by *C. roseus*, along with their “non-natural” derivatives [2], are potent chemotherapy agents used in the treatment of leukemia, lymphoma, and other solid tumors [3,4]. Industrial production of these pharmaceuticals currently relies on the extraction of the precursor monomers, catharanthine and vindoline, from the *C. roseus* plant, followed by their chemical coupling in vitro [5]. However, the low abundance of these precursors in plant tissues (milligrams per gram dry weight) contributes substantially to the high cost of vinca-based drugs [6]. Moreover, rising global demand has led to recurrent supply shortages, exacerbated by socioeconomic pressures, geopolitical instability, and climate-related constraints [7]. Thus, metabolic engineering and synthetic biology in either homologous or heterologous systems offer promising strategies to increase the production of vinblastine and its precursors, thereby reducing costs and helping to meet clinical needs more sustainably.

With the advancement of synthetic biology and the full elucidation of the monoterpene indole alkaloid (MIA) biosynthetic pathway, microorganisms, particularly yeast, have emerged as promising heterologous hosts for producing these compounds [8]. Notably, Zhang et al. [9] and Gao et al. [10] have achieved the de novo biosynthesis of catharanthine, vindoline, and even vinblastine in yeast. Despite these breakthroughs, production levels in microbial systems remain substantially low—at least two orders of magnitude lower than those found in plants—and are still far from meeting industrial requirements. Further yield improvement is severely constrained by the suboptimal performance of plant-derived enzymes in heterologous hosts, namely, poor expression, low activity, and insufficient substrate specificity, as well as the significant metabolic burden imposed by the introduction of the heterologous pathway [11,12,13,14]. Given this, plants and plant tissues continue to serve as competitive platforms for producing these complex alkaloids [15]. Among these, hairy root systems—induced by *Agrobacterium rhizogenes*—offer notable advantages, such as genetic stability, rapid growth, consistent metabolite production, and scalability [16,17,18]. Moreover, hairy roots accumulate catharanthine, a key precursor of vinblastine, at levels comparable to those in seedlings, positioning them as one of the primary bioreactors for catharanthine biosynthesis [5,19].

Previous metabolic engineering strategies in *C. roseus* hairy roots have primarily focused on engineering upstream pathway genes to “push” metabolic flux, overexpressing terminal steps or transporters to “pull” flux toward the target compound, or manipulating global transcription factors. However, over the past years, quantitative data from *C. roseus* hairy root studies reveal highly variable outcomes. Overexpression of upstream indole (*anthranilate synthase*, *tryptophan decarboxylase*) [20] or iridoid (*geraniol-10-hydroxylase*, *1-deoxy-D-xylulose synthase*) [21] pathway genes perturbed the accumulation of Corynanthe and Aspidosperma MIAs, but catharanthine levels remained largely unchanged. While manipulating transcription factors like *CrMYC1* [22] and *ORCA2* [23] improved catharanthine accumulation by 3- to 4.4-fold and ~0.3-fold, respectively, others such as *ORCA3* or *WRKY1* resulted in negative outcomes, highlighting the unpredictability of global regulatory networks [24]. Combining overexpression of *ORCA3* and the pathway gene *strictosidine glucosidase* led to a 38% increase in catharanthine level in *C. roseus* hairy roots [25]. Alternatively, overexpressing *catharanthine transporter* (*CrTPT2*) created a physical “sink” that dramatically increased accumulation by up to five-fold [26]. However, relying on spatial transport does not fundamentally rewire the biosynthetic bottlenecks within the cell.

A noticeable challenge in metabolic engineering of the MIA pathway is the presence of multiple branch points that divert flux toward non-target competitive alkaloids—such as ajmalicine, tabersonine, vincamine, and lochnericine—resulting in a dispersed secondary metabolic flow [27]. Branch-node engineering has emerged as an effective strategy to strictly route secondary metabolites in plant systems including *Nicotiana benthamiana* [28] and *C. roseus* [29]. In *C. roseus*, overexpressing the upstream branch-point gene *geranyl diphosphate synthase* (*GPPS*) significantly enhanced catharanthine by 1.5- to 1.7-fold in T_0_ transgenic plants [29]. Moving downstream to the highly competitive mid-to-late MIA pathway, geissoschizine synthase (GS) controls a major flux-directing node, competing with heteroyohimbine synthases (HYS) and tetrahydroalstonine synthase (THAS) [30]. Further downstream, catharanthine synthase (CS) governs the terminal branch point, competing with hydrolases (HL2, HL3, HL4) [31]. Previous reports have achieved transient expression of *GS* and *CS* alongside other late-stage genes in *N. benthamiana*, yielding a baseline of 60 ng/g frozen tissue, which highlighted GS as a critical flux-controlling enzyme [32].

However, the targeted diversion of metabolic flux at these mid-to-late branch points has not been systematically investigated in the native *C. roseus* chassis. Traditional upstream engineering often struggles to sustainably elevate specific downstream products, as competitive branches continuously dilute the “pushed” metabolic flux. To address this research gap, our study introduces a dual branch-point diversion strategy targeting the mid-to-late MIA pathway by overexpressing *GS* and *CS* in *C. roseus* hairy roots. By simultaneously securing both the mid-pathway entry node and the terminal exit node, this strategy yielded a 67% elevation (achieving up to 1.98 mg/g DW) in catharanthine accumulation. Furthermore, to explore the underlying mechanisms, we systematically investigated the impact of this dual diversion on broader MIA profiles and the transcriptional changes of pathway genes and associated regulatory factors.

## 2. Results

### 2.1. Generation of GS/CS Transgenic Hairy Roots

**Figure 1:**
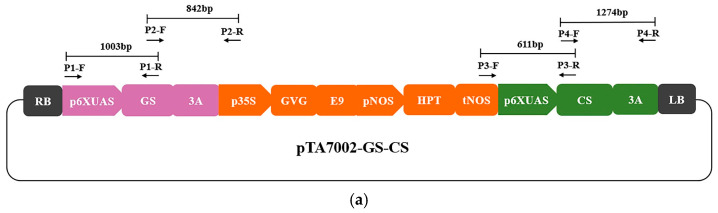

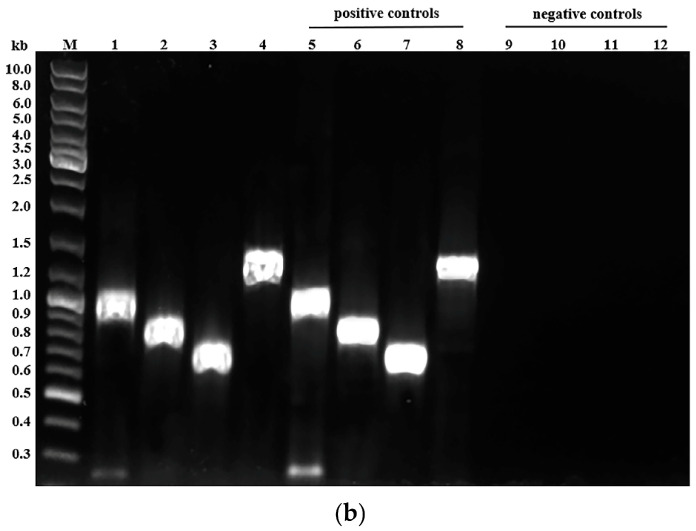
(**a**) Schematic representation of the plasmid construct used for *C. roseus* hairy root transformation and the primer binding sites for transgene verification. *RB*, right border of T-DNA; *LB*, left border of T-DNA; *p35S*, cauliflower mosaic virus 35S promoter; *GVG*, chimeric transcription factor containing GAL4 DNA-binding domain and VP16 transactivating domain and rat-GR HBD; *E9*, pea rbcS-E9 polyadenylation sequence; *pNOS*, nopaline synthase promoter; *HPT*, hygromycin phosphotransferase gene; *tNOS*, nopaline synthetase polyadenylation sequence; *p6xUAX*, GVG-regulated promoter; and *3A*, pea rbcS-3A polyadenylation sequence. (**b**) Genomic PCR verification of transgenic hairy roots. Lanes 1–4: PCR products amplified from transgenic hairy root genomic DNA amplified with primer sets 1, 2, 3, and 4, respectively, as shown in (**a**); Lanes 5–8: positive controls (plasmid template); Lanes 9–12: PCR products from wild-type hairy root genomic DNA (negative control).

*C. roseus* seedlings were transformed using *A. rhizogenes* ATCC15834 harboring the binary vector pTA7002*/GS/CS*. Following infection, induced hairy roots were excised and selectively cultured on solid medium supplemented with 300 mg/L cefotaxime and 30 μg/mL hygromycin. Four vigorously growing hairy root lines were subsequently chosen for subculture and adaptation to liquid medium. Of the four initially selected hairy root lines, three were excluded due to suboptimal growth and inconsistent performance. The remaining line, which demonstrated rapid biomass accumulation and stable liquid–culture adaptation, was successfully established for subsequent genetic validation and metabolic profiling. Genomic DNA extracted from the selected line was subjected to PCR using primers targeting the *GS* and *CS* transcription units. Positive amplification of both fragments confirmed successful genetic transformation (Figure 1).

**Figure 2:**
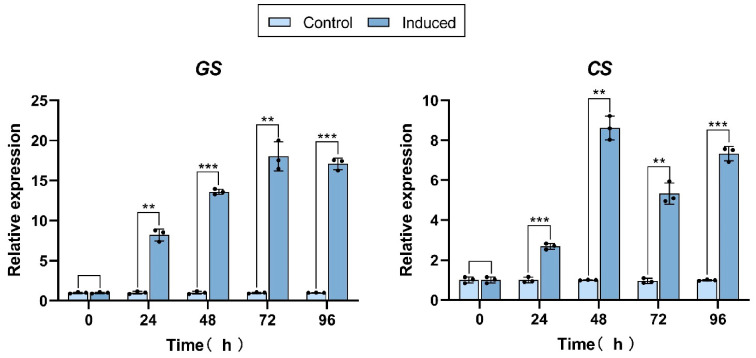
Gene expression levels of *GS* and *CS* in *C. roseus* transgenic hairy roots. The hairy roots overexpressing *GS* and *CS* were induced with 3 μM dexamethasone for 0, 24, 48, 72, and 96 h. The control was the same root line fed with an equivalent volume of ethanol. Error bars represent the standard deviation of three replicates. Statistical significance was determined by one-way ANOVA followed by Tukey’s HSD test, with ** indicating *p* < 0.01, and *** indicating *p* < 0.001.

Both *GS* and *CS* genes were placed under the control of a glucocorticoid-inducible promoter, enabling us to investigate the effects of induced expression relative to the control within the same genetic background [33]. This system also allows growth to be separated from secondary metabolite production. Hairy roots subcultured in liquid medium for 18 days were treated with 3 μM dexamethasone for 0, 24, 48, 72, or 96 h to induce gene expression; control samples received an equal volume of ethanol. RT-qPCR analysis revealed that dexamethasone induction for 72 h resulted in an 18-fold increase in *GS* mRNA levels compared to the control, whereas extending the induction to 96 h did not lead to further elevation. Meanwhile, *CS* mRNA peaked at 48 h post-induction, showing an 8.6-fold enhancement (Figure 2).

### 2.2. MIA Accumulation in Transgenic C. roseus Hairy Roots

**Figure 3:**
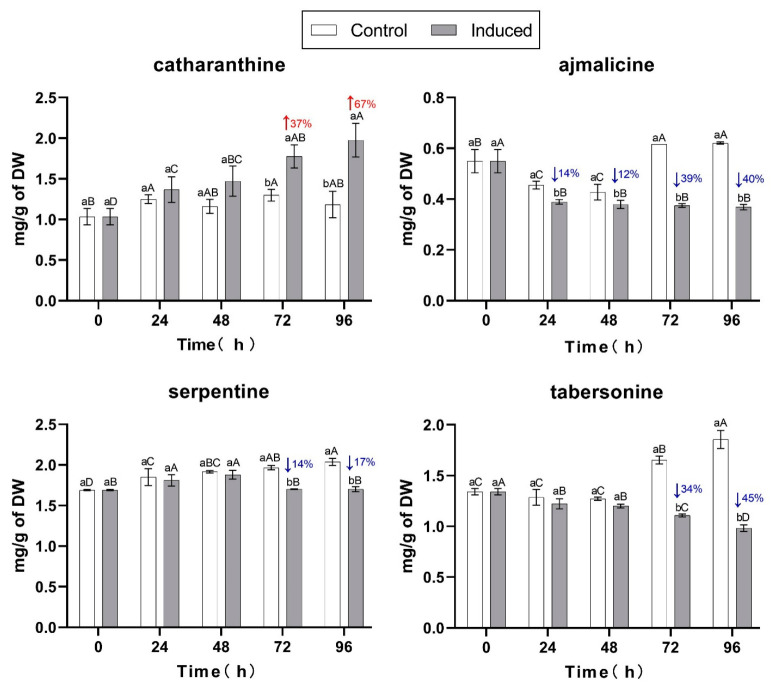
Alkaloid content in transgenic *C. roseus* hairy roots under induced and control conditions for different time periods. Statistical significance of differences in alkaloid content was determined by two-way ANOVA followed by Tukey’s HSD test (*p* < 0.05). Capital letters denote significant differences across time points within the same treatment group, whereas lowercase letters indicate significant differences between treatment groups at the same time point.

Catharanthine, the target metabolite of this study, serves as a direct precursor for the synthesis of vinca alkaloid anticancer drugs and is a critical raw material in their industrial production. Following the overexpression of *GS* and *CS* in *C. roseus* hairy roots for different durations, catharanthine and some competing alkaloids in the MIA pathway were quantified by HPLC. The calibration curves used for HPLC quantification are presented in Supplementary Figure S1. Compared with the control, catharanthine content did not increase significantly at 24 h or 48 h after induction. However, after 72 h and 96 h of induction, catharanthine levels were elevated significantly by 37% and 67%, respectively. The highest catharanthine concentration (1.98 ± 0.21 mg/g DW) was observed after 96 h of induction. Conversely, co-overexpression of *GS* and *CS* resulted in reduced accumulation of ajmalicine and serpentine, both derived from the upstream competing pathway. Ajmalicine levels declined progressively, decreasing by 14%, 12%, 39%, and 40% at 24 h, 48 h, 72 h, and 96 h, respectively, relative to the control. Serpentine followed a similar trend, remaining unchanged at 24 h and 48 h before decreasing by 14% and 17% at 72 h and 96 h. Tabersonine and its derivatives are classified as aspidosperma-type alkaloids. The tabersonine-derived branch constitutes another competing pathway for catharanthine biosynthesis, wherein tabersonine synthase (*HL2*) competes for the precursor dihydroprecondylocarpene acetate. As shown in Figure 3, tabersonine content did not differ significantly from the control at 24 h or 48 h post-induction. However, upon prolonged induction to 72 h and 96 h, tabersonine production decreased by 34% and 45%, respectively. Collectively, these findings demonstrate that co-overexpression of *GS* and *CS* reduces the accumulation of metabolites in competing branches, concomitantly enhancing catharanthine production.

### 2.3. Effect of GS and CS Overexpression on MIA Pathway Genes

Overexpression of MIA pathway genes typically triggers complex transcriptional responses across the pathway [34]. To evaluate the metabolic engineering strategy for catharanthine production at the transcriptional level, we examined the expression of MIA biosynthetic genes and regulators in response to *GS* and *CS* overexpression in *C. roseus* hairy roots.

**Figure 4:**
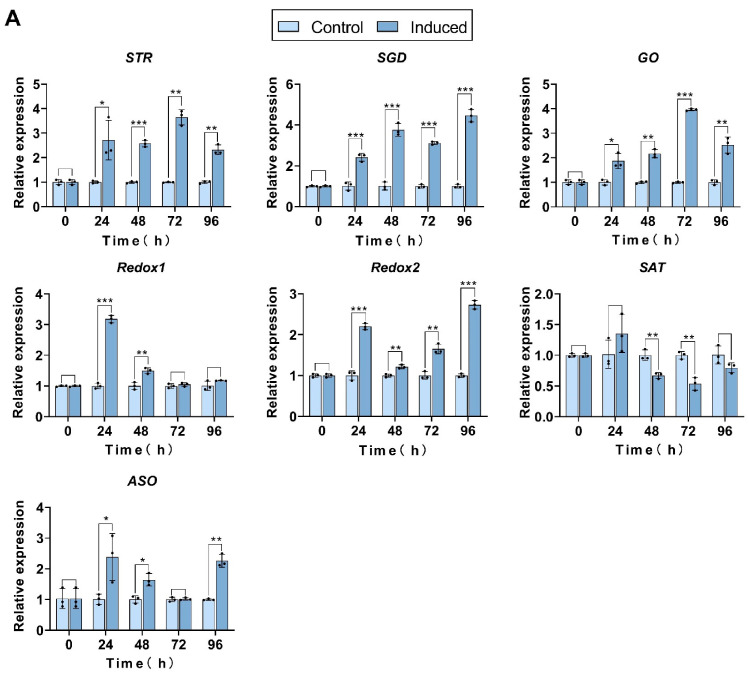

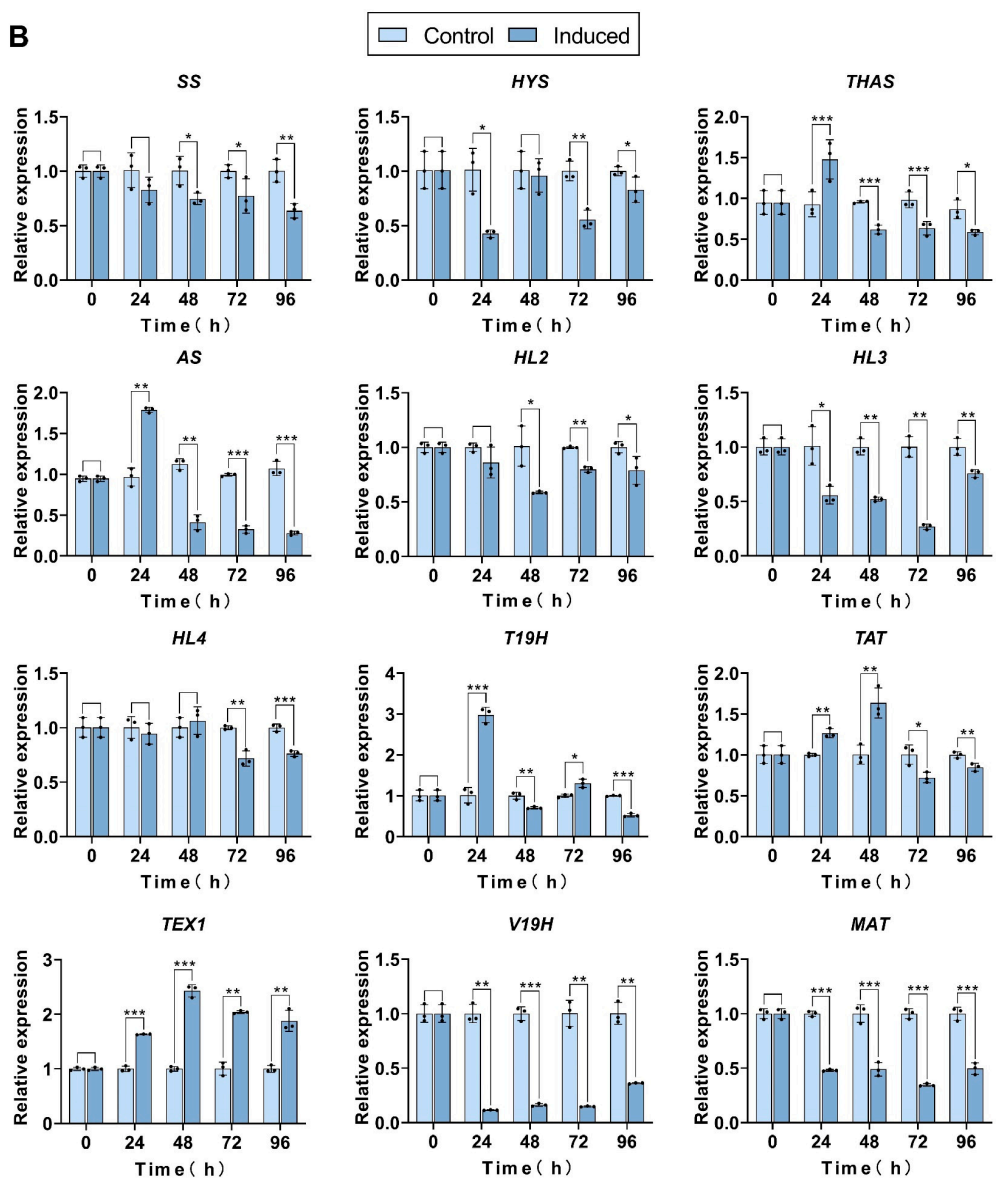
Relative mRNA levels of MIA pathway genes in hairy roots following induction of *GS* and *CS* overexpression with 3 μM dexamethasone for 0, 24, 48, 72, and 96 h compared with the control (the same root line treated with an equivalent volume of ethanol). (**A**) Relative expression of MIA pathway genes involved in catharanthine biosynthesis. (**B**) Relative expression of genes involved in pathways that compete with catharanthine biosynthesis. Error bars represent the standard deviation of three biological replicates. Statistical significance was analyzed by one-way ANOVA followed by Tukey’s HSD test. Asterisks indicate statistical significance: * *p* < 0.05, ** *p* < 0.01, *** *p* < 0.001.

*Strictosidine synthase* (*STR*) and *strictosidine β-D-glucosidase* (*SGD*), located upstream of *GS*, were upregulated at all four induction time points examined (Figure 4A). *STR* showed peak upregulation (3.6-fold) at 72 h induction, while *SGD* mRNA levels reached their maximum increase (103%) at 96 h (Figure 4A). Among the enzymes catalyzing conversions between *GS* and *CS*, distinct expression patterns emerged. *Geissoschizine oxidase* (*GO*) expression increased progressively, peaking at 72 h before declining at 96 h. *Reductase 2* (*Redox2*) expression reached its highest level at 96 h (2.8-fold relative to control). *O-acetylstemmadenine oxidase* (*ASO*) was upregulated at 24 h, 48 h, and 96 h, but showed no significant change at 72 h. (*Reductase 1*) *Redox1* exhibited sharp upregulation (3.2-fold) at 24 h that weakened by 48 h induction and became non-significant at later induction periods (Figure 4A). Notably, *stemmadenine-O-acetyl transferase* (*SAT*) expression was significantly downregulated (0.7-fold at 48 h and 0.5-fold at 72 h), suggesting that it may represent a bottleneck in catharanthine accumulation following *GS* and *CS* co-overexpression (Figure 4A).

In the upper competitive branches of catharanthine biosynthesis, *tetrahydroalstonine synthase* (*THAS*), *alstonine synthase* (*AS*), *heteroyohimbine synthase* (*HYS*), and *serpentine synthase* (*SS*) expression was altered by *GS* and *CS* overexpression. *THAS* and *AS*, which encode enzymes involved in alstonine biosynthesis [27,35], showed transient significant increases at 24 h followed by progressive downregulation at later time points. By 96 h, their expression reached minimal levels (0.6-fold for *THAS* and 0.3-fold for *AS* relative to controls) (Figure 4B). *HYS* and *SS*, involved in ajmalicine and serpentine biosynthesis, respectively, exhibited consistently lower expression in induced root lines throughout most induction periods. The strongest suppression occurred for *HYS* at 24 h (0.4-fold of control) and for *SS* at 96 h (0.6-fold of control) (Figure 4B), suggesting that reduced ajmalicine and serpentine production results from coordinated downregulation of these branch pathway genes.

*HL2/TS*, *HL3*, and *HL4* encode enzymes in competing pathways that divert intermediates toward tabersonine and vincadiformine production [31,36]. RT-qPCR analysis showed *HL2/TS* expression was significantly downregulated in induced roots at 48, 72, and 96 h, correlating with decreased tabersonine levels observed at 72 and 96 h. Similarly, *HL3* expression was significantly lower in induced roots throughout all tested periods, reaching its minimum (0.3-fold of control) at 72 h (Figure 4B). The downstream MIA pathway genes *tabersonine6*,*7-epoxidase* (*TEX1)*, *tabersonine19-hydroxylase* (*T19H)*, *tabersoninederivative19-O-acetyltransferase* (*TAT)*, *vincadifformine19-hydrolase* (*V19H)*, and *minovincine19-hydroxy-O-acetyltransferase* (*MAT)* convert tabersonine or vincadiformine to root-specific alkaloids such as hörhammericine, minovincinine, and echitovenine [37,38]. *TEX1* mRNA levels remained consistently elevated in induced roots compared to controls. In contrast, *T19H* and *TAT* displayed complex expression patterns. *T19H* was upregulated at 24 h and 72 h (3.0-fold and 1.3-fold, respectively) but downregulated at 48 h and 96 h (0.7-fold and 0.5-fold of control, respectively). Similarly, *TAT* expression increased at 24 h and 48 h (1.3-fold and 1.6-fold, respectively) but decreased at 72 h and 96 h (0.7-fold and 0.8-fold of control, respectively) (Figure 4B). *V19H* and *MAT*, which catalyze the conversion of vincadiformine to echitovenine, showed significantly lower expression in induced roots across all time points, indicating downregulation of this downstream branch pathway in response to *GS* and *CS* overexpression.

### 2.4. Effect of GS and CS Gene Overexpression on MIA Pathway Regulators

**Figure 5:**
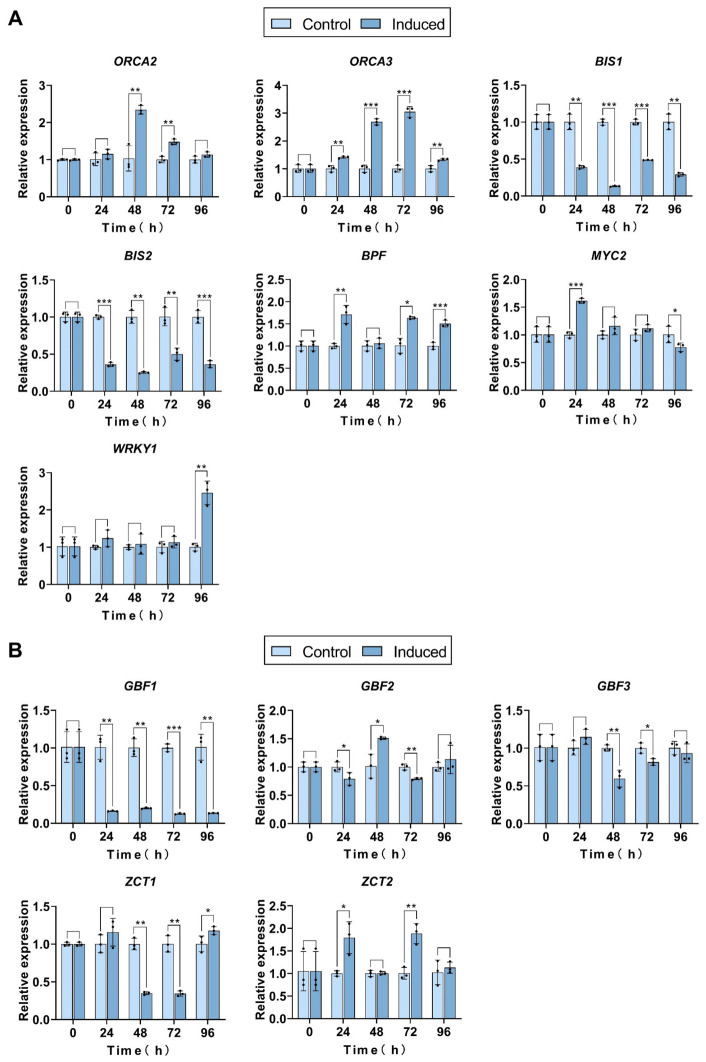
Relative mRNA levels of MIA pathway regulators in hairy roots following induction of *GS* and *CS* overexpression with 3 μM dexamethasone for 0, 24, 48, 72, and 96 h compared with the control (the same root line treated with an equivalent volume of ethanol). (**A**) Relative expression of MIA-positive regulators. (**B**) Relative expression of MIA-negative regulators. Error bars represent the standard deviation of three biological replicates. Statistical significance was analyzed by one-way ANOVA followed by Tukey’s HSD test. Asterisks indicate statistical significance: * *p* < 0.05, ** *p* < 0.01, *** *p* < 0.001.

The MIA pathway in *C. roseus* is tightly regulated by multiple transcription factors. In this study, qPCR was used to analyze the effects of overexpressing *GS* and *CS* genes on the expression of key transcriptional regulators of the MIA pathway. *ORCA2* and *ORCA3*, MeJA-responsive AP2/ERF transcription factors [39,40], were significantly upregulated following the induction of *GS* and *CS* overexpression, with maximum upregulation observed at 48 h and 72 h post-induction, respectively. MYC2, a bHLH transcription factor that can activate the MIA pathway either indirectly by regulating ORCA expression or directly by binding to key pathway genes, such as *TDC* [41], showed variable expression after *GS* and *CS* overexpression. It was upregulated at 24 h post-induction but downregulated at 96 h. WRKY transcription factors are also responsive to MeJA. *WRKY1*, which is preferentially expressed in roots and may regulate the serpentine biosynthetic pathway by activating *TDC* [42], showed unchanged mRNA levels until 72 h after induction. By 96 h, its expression was significantly elevated, suggesting that WRKY1 acts as a late-responsive factor following *GS* and *CS* overexpression. In contrast, *BIS1* and *BIS2*, MeJA-responsive bHLH transcription factors that regulate secoiridoid pathway genes [43], were largely downregulated at all time points. This downregulation may limit secoiridoid precursor supply for downstream MIA biosynthesis. Additionally, *BPF-1*, which may function through JA-independent elicitor signaling [44], was upregulated at 24, 72, and 96 h post-induction (Figure 5A).

The mRNA levels of the negative regulators showed mixed results in response to overexpressing *GS* and *CS.* The *GBFs* are reported to act as transcriptional repressors of MIA biosynthetic genes by antagonizing MYC2, thereby fine-tuning the MIA pathway [45,46]. Among them, *GBF1* was markedly downregulated to 0.1–0.2 times the control level across all induction periods. *GBF3* showed a similar trend, except that its expression remained unchanged after 96 h of induction. *GBF2* expression showed a decline at 24 and 72 h post-induction and an increase after induction for 48 h relative to the control. ZCTs are zinc finger transcription repressors in *C. roseus* that can be activated by MYC2 and ORCA proteins [47]. As shown in Figure 5B, *ZCT1* was significantly downregulated at 48 and 72 h after induction, reaching its lowest expression (0.3-fold of the control) at 72 h. With induction of the overexpression prolonged to 96 h, it was upregulated. In contrast, *ZCT2* showed significant upregulation at 24 and 72 h post-induction, with no notable changes at other time points. These mixed results suggest that MIA biosynthesis is fine-tuned and subject to intricate regulation.

### 2.5. Metabolomic Analysis and Differential Metabolites

**Figure 6:**
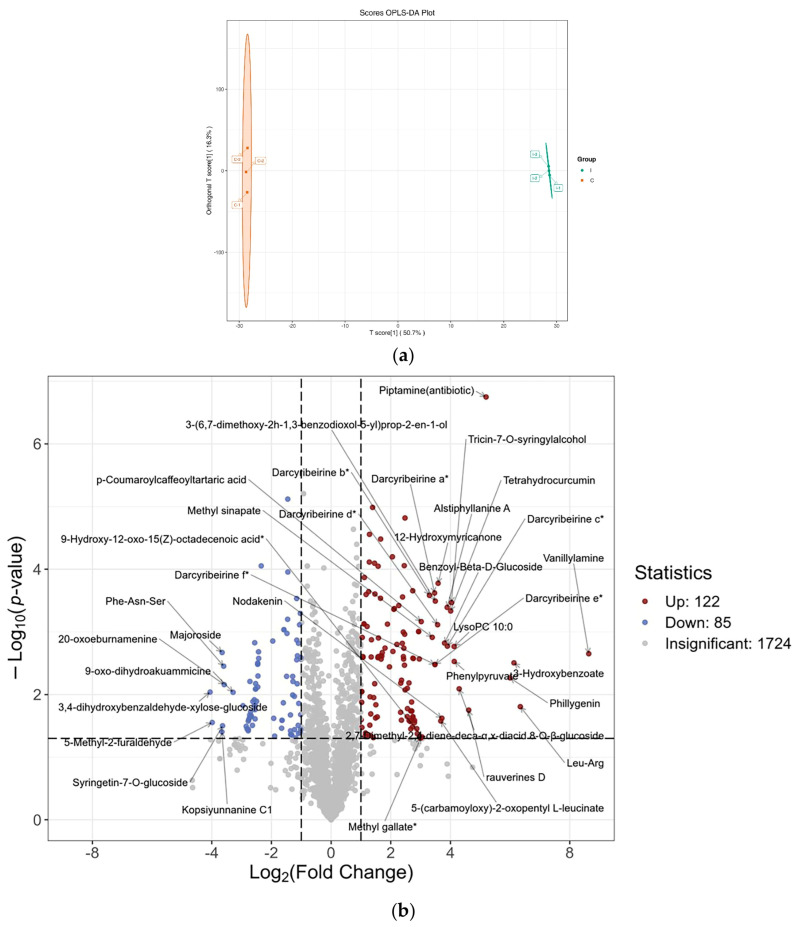
Metabolomic profiling of transgenic *GS* and *CS* hairy roots at 72 h post-induction compared with the uninduced controls. (**a**) OPLS-DA score plot showing distinct clustering between the two *C. roseus* hairy root groups. (**b**) Volcano plot showing differentially abundant metabolites in *GS* and *CS* overexpressing hairy roots compared with the control. * denotes isomers that co-elute and share identical MS/MS spectra.

To further investigate the global metabolic profile changes in response to the co-overexpression of *GS* and *CS* in *C. roseus* hairy roots, we performed UPLC-MS/MS-based metabolomic analysis on induced and uninduced transgenic hairy roots. Metabolomics samples were collected at 72 h—the time point when catharanthine accumulation started to increase significantly (Figure 3)—to optimally capture transient intermediates during the active phase of metabolic flux, prior to the final product peaking at 96 h. A total of 1724 metabolites were identified by comparing precursor ions with their corresponding fragment ions from the local metabolomics dataset. The relative abundance of these metabolites was subsequently analyzed using MetaboAnalyst. A noticeable separation between the metabolite profiles of induced and uninduced groups was observed in the OPLS-DA score plot (Figure 6a). To ensure the reliability of this supervised model and rule out overfitting, a 200-iteration permutation test was performed. The results demonstrated robust predictive ability (R^2^Y = 1, Q^2^ = 0.935, Q^2^ intercept < 0; Supplementary Figure S2), and the group separation was fundamentally supported by unsupervised Principal Component Analysis (PCA). Validated by these metrics, the data conclusively indicate that co-overexpressing *GS* and *CS* genes led to significant metabolic shifts. Compared with the uninduced control, 122 metabolites were upregulated and 85 were downregulated in the *GS* and *CS* overexpressing group (Figure 6b).

**Figure 7:**
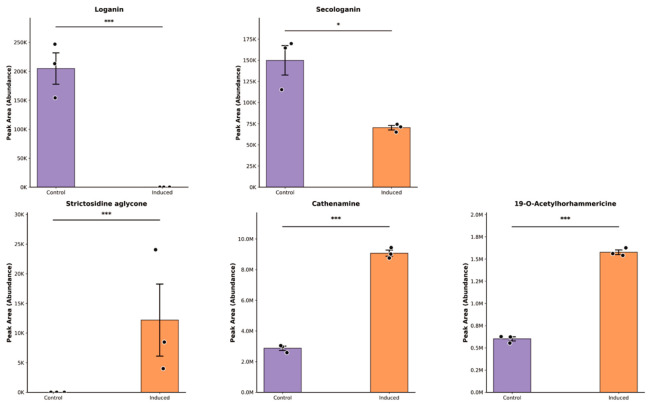
Levels of differentially accumulating high-abundant MIAs are expressed in the peak area of total ion current (TIC). Statistical significance is indicated by * *p* < 0.05, *** *p* < 0.001.

Metabolic profiling revealed that the top three most significantly increased metabolites were vanillylamine, 3-hydroxybenzoate, and phillygenin, all of which belong to aromatic amino acid-derived secondary metabolites. These compounds share an indole precursor synthesized via the shikimate pathway, underscoring a close connection between the MIA pathway and other aromatic amino acid-derived secondary metabolic routes. However, the direct indole precursors for MIA biosynthesis, tryptophan and tryptamine, showed no significant changes following *GS* and *CS* overexpression. Intriguingly, the terpenoid precursors loganin and secologanin were significantly reduced. Among the altered metabolites, three MIA-pathway intermediates, including strictosidine aglycone, cathenamine, and 19-O-acetylhorhammericine, were strongly upregulated (Figure 7). The first two are intermediates in catharanthine biosynthesis, while the latter is the end product of a root-specific downstream branch of the MIA network that competes with catharanthine biosynthesis for the shared precursor dihydroprecondylocarpine acetate. Together with the HPLC results (Figure 3), these data indicate that although upstream terpenoid biosynthesis may be constrained, overexpression of *GS* and *CS* systemically reprograms the metabolic profile to favor the downstream MIA biosynthetic pathway.

**Figure 8:**
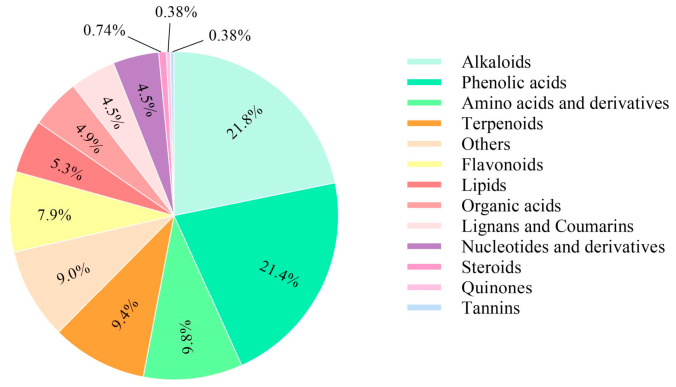
Categorization of differentially abundant metabolites in *GS* and *CS* co-overexpressing hairy roots upon induction relative to uninduced controls.

Subsequent classification of the differential metabolites was performed to evaluate their category distribution between the induced overexpression group (72 h) and the uninduced control group (72 h). As shown in Figure 8, co-overexpression of *GS* and *CS* genes shifted the metabolic profile from primary to secondary metabolites. The altered metabolites predominantly belonged to secondary metabolite categories, including alkaloids, phenolic acids, terpenoids, flavonoids, lignans and coumarins, steroids, quinones, and tannins. Notably, the alkaloid group constituted the largest proportion (21.8%) of the total differential metabolites (Figure 8). Primary metabolite categories, such as lipids, amino acids, and nucleotides, were also perturbed, indicating a complex and systematic metabolic alteration in response to *GS* and *CS* co-overexpression.

**Figure 9:**
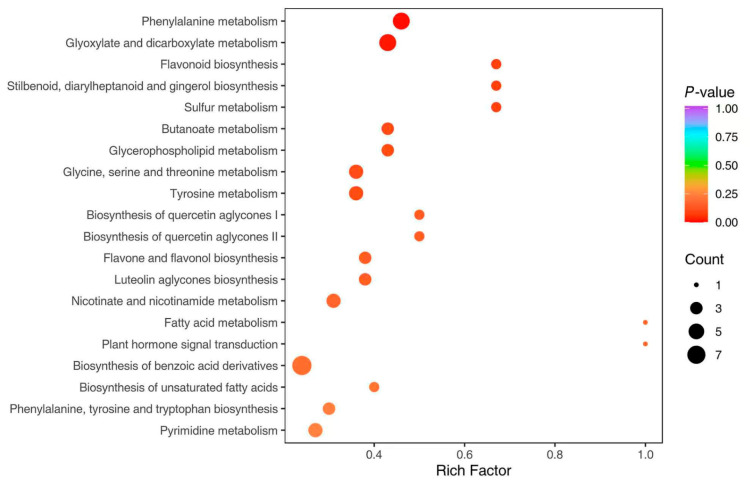
Top 20 significantly enriched KEGG pathways (*p* < 0.05) in induced vs. uninduced GS and CS co-overexpressing hairy roots, ranked by *p*-value. Rich Factor, metabolite count (bubble size), and *p*-value (color) are indicated.

KEGG pathway enrichment analysis of the differentially accumulated metabolites in the induction group (Figure 9) revealed their significant association with plant hormone signal transduction and fatty acid biosynthesis. Overexpression of *GS* and *CS* also perturbed several secondary metabolic pathways, including flavonoid biosynthesis, stilbenoid, diarylheptanoid, and gingerol biosynthesis, and phenylalanine metabolism. Furthermore, induction of transgene expression affected multiple primary metabolic pathways, such as fatty acid metabolism, sulfur metabolism, and glyoxylate and dicarboxylate metabolism.

## 3. Discussion

### 3.1. Overexpression of GS and CS Shifted the Metabolic Profile Toward Catharanthine Accumulation

Up to now, numerous metabolic engineering strategies have been studied in *C. roseus* plants, cell cultures, and hairy root systems. Most strategies targeted the upstream MIA pathway genes or regulators, yielding limited success in increasing catharanthine production [24,48]. Because catharanthine biosynthesis via the MIA pathway involves several competing branches leading to by-products, including corynanthe-type metabolites (serpentine, ajmalicine, alstonine) and aspidosperma-type metabolites (tabersonine and its derivatives), little effort has been devoted to reallocating metabolic flux at these branch points. In this study, we applied a metabolic engineering strategy involving the co-overexpression of *GS* and *CS* genes at the key catharanthine biosynthesis branch node in *C. roseus* hairy roots to maximize catharanthine accumulation.

The successful induction of *GS* and *CS* overexpression, confirmed by elevated *GS* and *CS* transcript levels, demonstrates the establishment of functional transgenic *C. roseus* hairy root lines under the control of a glucocorticoid-inducible promoter. The substantial increases in catharanthine accumulation—by 37% and 67% after 72 and 96 h of *GS* and *CS* overexpression, respectively—indicate that metabolite accumulation was successfully shifted toward the target product. A temporal lag was observed between the transcript peaks of *CS* (48 h) and *GS* (72 h) and the maximum catharanthine accumulation (96 h). This delay primarily reflects the inherent time required for mRNA translation and enzyme maturation. Additionally, catharanthine accumulation is a cumulative process resulting from sustained enzymatic activity following the transcription peaks. Given the complexity of the MIA pathway, upstream precursors generated by *GS* must undergo multiple intermediate enzymatic steps and subcellular trafficking before the terminal conversion by *CS*, collectively contributing to the delayed peak in end product abundance.

**Figure 10:**
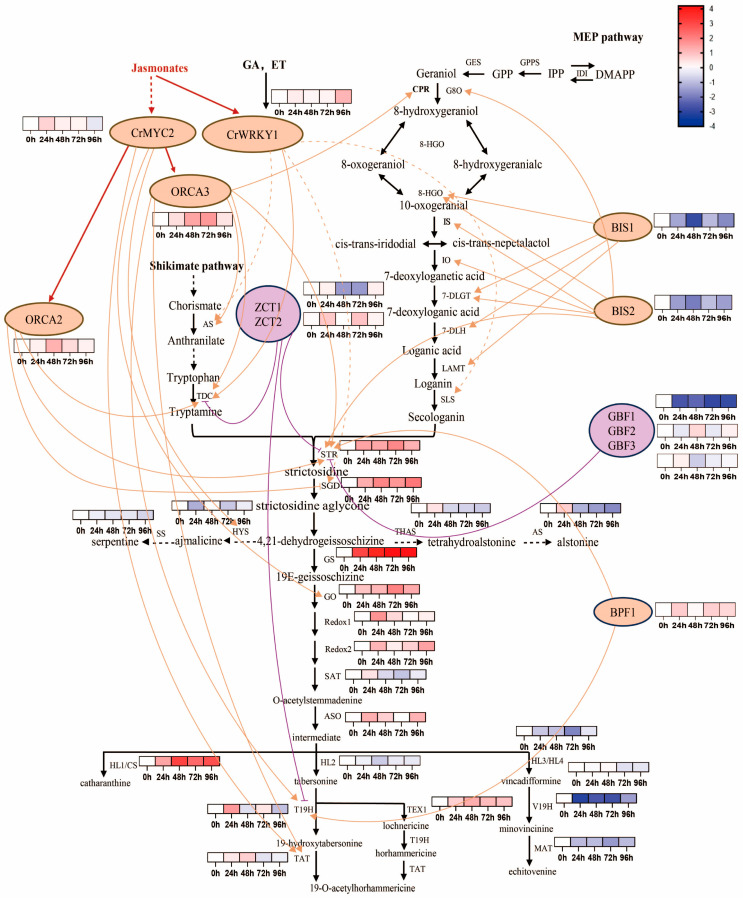
Integrative map of the MIA pathway and its transcriptional regulatory networks in *C. roseus*. Heatmaps adjacent to gene symbols display the temporal expression patterns (0, 24, 48, 72, and 96 h) in engineered hairy roots following the induction of *GS* and *CS* overexpression. Ovals represent key transcription factors. Orange solid and dashed arrows denote direct and indirect activation, respectively, while bar-headed purple lines indicate repression. Purple solid circles indicate negative regulators; orange circles indicate positive regulators.

Further qPCR analysis revealed that key MIA pathway genes involved in catharanthine biosynthesis (*STR*, *SGD*, *GO*, *redox1*, *redox2*, and *ASO*) were upregulated at multiple induction periods, collectively promoting flux into catharanthine synthesis (Figure 10). This was further corroborated by metabolomic data showing a significant increase in the levels of the intermediates strictosidine aglycone and cathenamine. Comparable engineering strategies have proven effective in other systems. For instance, overexpression of a branchpoint gene in the monoterpene biosynthesis pathway, the *geranyl diphosphate synthase small subunit 1* (*LcGPPS.SSU1*), substantially enhanced monoterpene production in *Litsea cubeba* and *Nicotiana benthamiana* [28]. In *C. roseus*, modification of the upstream branch point in the monoterpene biosynthetic pathway has been reported. Overexpression of the *GPPS*, which supplies precursors for the early steps of secologanin biosynthesis, significantly enhanced secologanin accumulation. Given that secologanin provides the monoterpenoid moiety for MIAs, this modification thereby elevated the levels of MIAs in certain T_0_ and T_1_ generation transgenic plants [29]. Our results build upon this concept by demonstrating that mid-to-late-stage branch-point engineering is equally, if not more, critical for specific alkaloid enrichment.

In parallel, overexpression of *GS* and *CS* triggered the downregulation of competing branch pathways. *HYS* and *SS*, which are responsible for the biosynthesis of corynanthe-type metabolites (serpentine and ajmalicine), and *HL2*, *HL3*, and *HL4*, for aspidosperma-type metabolites (tabersonine and vincadifformine), were downregulated during most induction periods (Figure 10). This downregulation alleviated competition for shared intermediate substrates at two branch points, thereby favoring flux toward catharanthine and explaining the observed decrease in serpentine, ajmalicine, and tabersonine levels. Although the precise mechanistic driver for this downregulation remains unclear, it likely reflects a homeostatic metabolic shift, potentially associated with the depletion of immediate precursors or mediated by feedback regulatory networks to maintain intracellular homeostasis. Interestingly, the targeted overexpression also activated specific genes from downstream competing pathways. Notably, genes involved in a root-specific downstream pathway (*TEX1*, *T19H*, and *TAT*) were transiently upregulated, which accounts for the ~2.6-fold increase in the tabersonine-derived end product 19-O-acetylhorhammericine detected by metabolic profiling. Collectively, these results demonstrate that the MIA metabolic profile was successfully shifted toward the downstream branches. However, the concurrent induction of certain competing pathway genes may have partially constrained the reallocation of flux toward catharanthine biosynthesis. In addition, a limitation of the current study is the absence of stable single-gene overexpressing hairy root lines for GS or CS. Consequently, while our co-overexpression strategy yielded a significant 67% increase in catharanthine, we cannot definitively attribute this increment to a strictly synergistic mechanism rather than an additive effect. Future investigations incorporating individual transgenic lines will be necessary to fully uncouple the precise contributions of each enzyme.

### 3.2. Tight and Complex Regulation Limited the Increase in Catharanthine

Although the metabolic engineering strategy of co-overexpressing *GS* and *CS* successfully elevated catharanthine levels, the overall fold changes (1.37- to 1.67-fold relative to the control) remained limited. Transcriptional analysis of the MIA pathway revealed a transient burst in expression: nearly all genes involved in catharanthine biosynthesis were significantly upregulated during the early induction phase. However, this upregulation gradually attenuated for most mid-pathway genes, including *Redox1*, *Redox2*, *SAT*, and *ASO*, with *SAT* even becoming significantly downregulated after 48 h of induction. This dynamic response in mRNA levels indicates that catharanthine production is under the tight control of a complex regulatory system, where positive and negative regulators act synergistically to fine-tune MIA gene expression.

Indeed, most of these regulators themselves exhibited dynamic temporal shifts in response to *GS* and *CS* overexpression. The early-responsive positive transcription factors (TFs) *MYC2* and *BPF* were highly upregulated at 24 h post-induction, whereas the activation of *ORCAs* peaked later at 48 or 72 h. *WRKY*, by contrast, only responded after 96 h. Although these positive TFs all belong to the MeJA-responsive signaling cascade [49], they followed distinct temporal patterns upon *GS* and *CS* perturbation. Given that *MYC2* is known to trigger *ORCA* upregulation, the observed lag between *MYC2* and *ORCA* induction perfectly aligns with this established regulatory hierarchy [50]. The metabolic shifts observed in our induced line might be related to compensatory feedback mechanisms. Previous findings indicate that specific transcription factors regulate distinct MIA pathway branches [51]. It is plausible that the artificial overexpression of branch-point enzymes (GS and CS) alters the pathway equilibrium, thereby prompting downstream regulatory adjustments. However, further studies using multiple transgenic lines and targeted knockouts are required to elucidate the exact mechanisms.

The limitation in the overall fold-increase in catharanthine might be related to a potential depletion of upstream terpenoid precursors, governed by the recalibrated regulatory network. While direct indole precursors remained stable, metabolomic profiling highlighted a significant reduction in loganin and secologanin. Intriguingly, this precursor depletion coincides with the persistent downregulation of *BIS1* and *BIS2*, the master transcriptional activators of the iridoid pathway. Recent evidence demonstrates that the efficient upregulation of the iridoid pathway requires the cooperative interaction between ethylene-signaling components and JA-induced *BIS* factors [52]. In our system, the robust metabolic “pull” generated by *GS* and *CS* overexpression could potentially alter these signaling dynamics, which might be associated with the suppression of *BIS* genes and the subsequent starvation of the terpenoid scaffold. Therefore, future metabolic engineering endeavors should pivot towards combinatorial strategies. Coupling the directed downstream flux with a synchronized “push” from upstream precursor pathways—such as overexpressing *BIS* regulators or key MEP pathway genes—could alleviate these precursor bottlenecks and fully unlock the biosynthetic potential of the engineered tissues.

### 3.3. Overexpression of GS and CS Triggered Global Metabolic Response from Primary to Secondary Pathways

**Figure 11:**
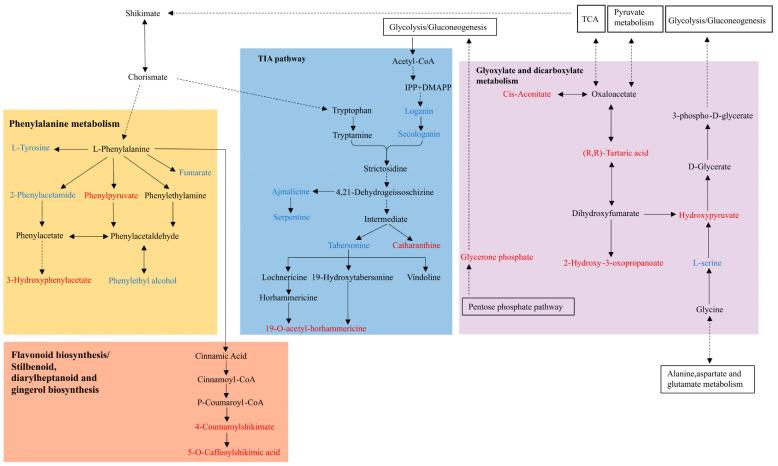
Significant enrichment of metabolic pathways and their association with MIA biosynthesis. Red and blue colors indicate significantly upregulated and downregulated metabolites after overexpressing *GS* and *CS*, respectively, compared to the control group.

Genetic perturbation of genes involved in MIA biosynthesis typically elicits a systematic response across the entire metabolic network, an effect strongly supported by our metabolic profiling data. MIA biosynthesis is intricately connected to primary metabolism through two key precursor streams: the indole pathway and the iridoid (terpenoid) pathway [53]. Figure 11 illustrates the most significantly enriched metabolic pathways and their relationship with MIA biosynthesis.

Both category and pathway enrichment analyses indicated that phenylalanine metabolism and phenylalanine-derived secondary pathways—including the biosynthesis of flavonoids, stilbenoids, diarylheptanoids, and gingerols—were highly impacted following the co-overexpression of *GS* and *CS* (Figure 11). Phenylalanine biosynthesis and the indole pathway are metabolically linked through their shared precursor, chorismate, which is derived from the shikimate pathway. Previous studies have demonstrated that, similar to the MIA pathway, phenylalanine-derived secondary pathways compete for this common precursor and can be simultaneously stimulated by various environmental factors or exogenous elicitors, such as methyl jasmonate (MeJA) and salicylic acid [50]. The observed fluctuations in phenylalanine metabolism and its downstream secondary pathways are consistent with findings from our earlier engineering work, in which manipulation of the indole pathway gene *AS* effectively reprogrammed phenylalanine metabolism [34]. Moving beyond simple carbon allocation, this metabolic shift toward phenylalanine-derived pathways may represent a compensatory mechanism to the metabolic stress induced by catharanthine or other MIA intermediates overproduction. Flavonoids and phenolic acids are well-documented for their roles in secondary metabolism buffering and stress signaling [54]. Their significant enrichment following *GS* and *CS* co-overexpression suggests a potential association between the altered MIA metabolic shifts and the activation of antioxidant pathways [55,56]. Rather than a targeted accommodation, this parallel upregulation might reflect a general stress response or a passive homeostatic adjustment mechanism within the engineered hairy roots. These observations align with the concept of metabolic plasticity in *C. roseus*, hinting that genetic perturbations in specific metabolic branches can coincide with shifts in broader protective pathways [57]. These results point to a possible regulatory and metabolic crosstalk between the MIA network and phenylalanine-derived pathways, a hypothesis that warrants further investigation and mechanistic validation. However, when interpreting these systemic responses, it should be noted that our untargeted metabolomics serves primarily as an exploratory screening based on relative quantification. Consequently, the observed perturbations in specific pathways, such as phenylalanine metabolism and secoiridoid biosynthesis, represent putative shifts, and future targeted LC-MS/MS analyses will be valuable to confirm their absolute quantitative changes.

Furthermore, the overexpression of *GS* and *CS* also induced significant global shifts in central carbon metabolism. The biosynthesis of iridoid precursors and other terpenoid scaffolds requires substantial amounts of foundational building blocks, specifically acetyl-CoA and malonyl-CoA [58], both of which are integrally involved in the glyoxylate and dicarboxylate metabolic pathways. Following co-overexpression, the glyoxylate and dicarboxylate metabolism pathway was significantly enriched—a critical process that facilitates the conversion of fatty acids into carbohydrates. This enrichment suggests that these central carbon metabolic pathways are upregulated to meet the escalated demand for shared biosynthetic precursors, thereby driving the observed fluctuations in the levels of intermediates within glyoxylate and dicarboxylate metabolism. It is important to note that the untargeted metabolomics data presented here represent a snapshot at the 72 h inflection point. While it successfully captures the peak global metabolic shift following gene induction, future time-series metabolomic studies will be needed to fully resolve the kinetic mechanisms of these pathway perturbations. In addition, while our glucocorticoid-inducible promoter design allows the uninduced state to serve as an effective isogenic internal control, thereby mitigating confounding variables related to T-DNA positional effects, our current metabolic profiling is fundamentally based on a single transgenic line. Therefore, while the induced-versus-uninduced comparisons are robust within this genetic background, they cannot fully substitute for the analysis of multiple independent transformation events. Future studies incorporating additional independent transgenic lines will be essential to fully capture broader line-to-line biological variability and to further substantiate these mechanistic claims.

## 4. Materials and Methods

### 4.1. Cloning Constructs

The plasmid pTA7002 was used for vector construction [59]. Total RNA was extracted from seedlings of *C. roseus* var. “Little Bright Eyes” and reverse-transcribed into cDNA using the PrimeScript™ RT Reagent Kit with gDNA Eraser (Takara Bio, Kusatsu, Shiga, Japan). The coding sequences of *GS* (Gene ID: MF770507.1) and *CS* (Gene ID: MF770512.1) were amplified using Q5^®^ High-Fidelity DNA Polymerase (New England BioLabs, Ipswich, MA, USA). Each fragment was independently cloned into the intermediate vector pUCGALA [60] using the ClonExpress^®^ MultiS One Step Cloning Kit (Vazyme Biotech., Nanjing, China). The *GS* fragment was excised from pUCGALA/*GS* using *Xho*I and *Spe*I (NEB) and ligated into *pTA7002* with T4 DNA ligase (Takara Bio) to generate *pTA7002/GS*. Subsequently, the *CS* expression cassette was cut from pUCGAL*A/CS using SbfI and inserted into* pTA7002*/GS to yield the co-expression vector* pTA7002/*GS/CS*. The correct orientation and sequence of both genes in the final construct were confirmed by sequencing. The resulting expression plasmid pTA7002*/GS/CS* was introduced into *Agrobacterium rhizogenes* strain ATCC 15834 via electroporation, and successful transformation was verified by sequencing.

### 4.2. C. roseus Hairy Root Generation, Culture, and Induction

*A. rhizogenes* ATCC 15834 harboring the co-expression plasmid pTA7002*/GS/CS* was cultured in 1 mL of YMB medium under controlled conditions (28 °C, 225 rpm) until the OD reached 0.5. Sterilized forceps were immersed in the bacterial suspension and subsequently used for stem puncture inoculation of *C. roseus* seedlings, following the established protocol described by Bhadra et al. [61].

Hairy roots were cultured as described previously [62]. Roots at the late exponential growth phase (18 days post-subculture) were divided into two groups: an induced group treated with 3 μM dexamethasone and an uninduced control group treated with an equivalent volume of ethanol. Samples were collected at 0, 24, 48, 72, and 96 h post-induction, with three biological replicates per group per time point, which refer to three independent culture and extraction replicates of the same clonal transgenic line.

### 4.3. Alkaloid Extraction and HPLC Analysis

Harvested roots were blotted dry, flash-frozen in liquid nitrogen, and lyophilized (Scientz-100F, Scientz, Ningbo, China). Approximately 50 mg of lyophilized tissue was homogenized and extracted with 30 mL of methanol via ultrasonication (Sonicator S4000, Misonix, Farmingdale, NY, USA) for 10 min. After centrifugation (6000 rpm, 15 min, 4 °C), the supernatants were concentrated to 2 mL via a vacuum evaporator (Labconco, Kansas City, MO, USA), filtered through 0.22 μm nylon membrane filters, and transferred to autosampler vials for HPLC analysis. Catharanthine, ajmalicine, serpentine, and tabersonine were purchased from TargetMol (Shanghai, China). These standards and samples were separated through an Agilent SB-C18 column (1.8 μm, 2.1 × 100 mm; Agilent Technologies, Santa Clara, CA, USA) on an HPLC system (SHIMADZU, Kyoto, Japan) as previously described [63]. Two-way ANOVA followed by Tukey’s HSD tests were used to evaluate the significant differences (*p* < 0.05).

### 4.4. RT-qPCR Amplification and Gene Expression Analysis

Total RNA was extracted from the fresh hairy root and reverse-transcribed into cDNA as described in the previous section. Each qPCR reaction contained 1 μL of diluted cDNA, 0.8 μL of primer mix, 10 μL of 2× SYBR qPCR Master Mix (Vazyme Biotech., Nanjing, China), and nuclease-free water. The primers used for qPCR are listed in Supplementary Table S1. Amplification was performed using the BIO-RAD CFX Connect™ real-time PCR detection system (Bio-Rad, Hercules, CA, USA) with the following program: 10 min at 95 °C, 40 cycles of 15 s at 95 °C, and 1 min at 60 °C [34]. The 40S ribosomal protein S9 (RPS9) was used as a reference gene to normalize for variations in gene expression and RNA extraction efficiency. One-way ANOVA followed by Tukey’s HSD tests were used to evaluate the significant differences (*p* < 0.05).

### 4.5. LC-MS Metabolomic Analysis

Metabolomics analyses were performed using three independent culture replicates for both the uninduced control and the 72 h induced groups. Sample processing and metabolomic analysis were performed by MetWare Co., Ltd. (Wuhan, China). Approximately 50 mg of powdered *C. roseus* hairy roots were extracted with 1.2 mL of 70% methanol. The sample was mixed by vortexing for 30 s every 30 min, for a total of 6 times, after which the sample was centrifuged at 12,000× *g* for 3 min. The resulting supernatant was filtered through a microporous membrane (0.22 μm pore size). A 10 µL aliquot from each sample was pooled to prepare a quality-controlled (QC) sample. Metabolite analysis was performed using an UPLC system (SHIMADZU Nexera X2, Kyoto, Japan) coupled with a tandem mass spectrometer (SCIEX P 6500 QTRA). Chromatographic separation was performed using an SB-C18 column (Agilent, USA; 100 mm × 2.1 mm, 1.8 μm) [64]. Mass spectrometry, metabolite identification, and quantification were performed following the standard procedures of Wuhan MetWare Biotechnology Co., Ltd., as described by Chen et al. [65].

The metabolite data were analyzed via orthogonal partial least squares discrimination analysis (OPLS-DA), cluster analysis, and Pearson’s correlation analysis using the R software package MetaboAnalystR 2.0 [66]. The metabolites identified through them were subjected to the OPLS-DA model [67]; differential metabolites were selected based on VIP (VIP > 1) and absolute Log_2_FC (|Log_2_FC| ≥ 1.0). Moreover, pathway enrichment analysis was performed using a custom Perl script implementing a hypergeometric test to evaluate whether the distribution of differentially accumulated metabolites across pathways deviated significantly from random expectation. The background set was defined as the union of all detected metabolites that were successfully annotated in the KEGG database (http://www.kegg.jp/kegg/pathway.html, accessed 8 July 2024). All detected metabolites were mapped to the general plant reference pathway. No minimum pathway size filter was applied; all pathways containing at least one background metabolite were included in the analysis. Raw *p*-values were calculated for each pathway and adjusted for multiple hypothesis testing using the Benjamini–Hochberg false discovery rate (FDR) procedure. Significant enrichment was defined using an exploratory threshold of raw *p* < 0.05 to preserve the discovery of biologically relevant metabolic shifts.

## 5. Conclusions

In summary, the targeted co-overexpression of *GS* and *CS* functions as a highly effective competitive branch-point diversion strategy rather than a simple downstream “pull.” By enhancing the catalytic dominance at these pivotal nodes, this approach successfully reduces intermediate accumulation in parallel alkaloid pathways while promoting catharanthine biosynthesis, accompanied by concurrent homeostatic adjustments across the metabolic network. However, even with the targeted enhancement of biosynthesis at these mid-to-late stages, total accumulation is ultimately constrained by the exhaustion of upstream terpenoid precursors and complex regulatory bottlenecks, notably the persistent downregulation of the *BIS* cluster. Therefore, future metabolic engineering endeavors must integrate this precise branch-point diversion with a synchronized upstream precursor “push” to fundamentally alleviate supply limitations and fully optimize *C. roseus* hairy root as a robust pharmaceutical chassis.

## Data Availability

The original contributions presented in this study are included in the article/Supplementary Material. Further inquiries can be directed to the corresponding author.

## Supplementary Materials

The following supporting information can be downloaded at: https://www.mdpi.com/article/10.3390/plants15142220/s1, Table S1: qPCR primers. Figure S1: The calibration curves used for HPLC quantification. Figure S2: OPLA-DA permutation.

## Abbreviations

The following abbreviations are used in this manuscript:

MIA	monoterpene indole alkaloid
GS	geissoschizine synthase
CS	catharanthine synthase
HYS	heteroyohimbine synthases
HL2	hydrolase 2
HL3	hydrolase 3
HL4	hydrolase
STR	strictosidine synthase
SGD	strictosidine β-D-glucosidase
GO	geissoschizine oxidase
Redox2	reductase 2
ASO	acetylstemmadenine oxidase
Redox1	reductase 1
SAT	stemmadenine-O-acetyl transferase
THAS	tetrahydroalstonine synthase
AS	alstonine synthase
SS	serpentine synthase
TEX1	tabersonine6,7-epoxidase
T19H	tabersonine19-hydroxylase
V19H	vincadifformine19-hydrolase
MAT	minovincine19-hydroxy-O-acetyltransferase
TAT	tabersoninederivative19-O-acetyltransferase
ORCA	AP2-domain DNA-binding protein
MYC2	MYC2 type basic helix–loop–helix transcription factor
BIS	B-box/iron sulfur protein
BPF1	B-box protein family 1
WRKY	WRKY transcription factor
ZCT	zinc finger Catharanthus transcription factor
GBF	G-box binding factor
KEGG	Kyoto Encyclopedia of Genes and Genomes
