# Supplementary material for: Co-Overexpression of Geissoschizine Synthase and Catharanthine Synthase Increases Catharanthine Biosynthesis in Catharanthus roseus Hairy Roots
Source: Plants (Basel). 2026 Jul 21;15(14):2220. doi: 10.3390/plants15142220 (PMC13416060; doi:10.3390/plants15142220)
Supplement: Supplementary file 1 [file plants-15-02220-s001.zip › Table S1. qPCR primers.pdf]

Supplementary Table S1. Primer sequences for validation by quantitative real-time PCR (qPCR).

| | |
|---|---|
| qPCR-GS-F | 5'-TTACCGTGAGCAAGGGGAAC-3' |
| qPCR-GS-R | 5'-TCCTGGCTTATCGAGTCCCA-3' |
| qPCR-CS-F | 5'-ACGACTCCAGATGAGACTAAA-3' |
| qPCR-CS-F | 5'-AACATATTCTTGCCCATCTCC-3' |
| qPCR-SGD-F | 5'-GGAACCTGGCAAAGAACCCT-3' |
| qPCR-SGD-R | 5'-CCCCTTTCCCGAGCATCAAT-3' |
| qPCR-STR-F | 5'-GTGAGAACAGCACCGATCCA-3' |
| qPCR-STR-R | 5'-TGTGTGGCATACCCACCTTC-3' |
| qPCR-THAS-F | 5'-AGTGGGCGTAGCAAGCATAA-3' |
| qPCR-THAS-R | 5'-TTCAGGCCAACGGATGACAA-3' |
| qPCR-AS-F | 5'-GAATCGGCCAACTTTCAGCC-3' |
| qPCR-AS-R | 5'-AGTAGTTTGTGGCCTGTCCG-3' |
| qPCR-HYS-F | 5'-GAAAAAGCGTGAAGCCCTCG-3' |
| qPCR-HYS-R | 5'-GGCTCCTCTGGTACCCCTAA-3' |
| qPCR-SS-F | 5'-AACTTTGCGGATAGGCCACA-3' |
| qPCR-SS-R | 5'-TCCGTGACATAAATTTGCCGC-3' |

| | |
|---|---|
| qPCR-GO-F | 5’-CGGGACCTTTACCTCATCGT-3’ |
| qPCR-GO-R | 5’-CGATCTGCAACGGCTAAACC-3’ |
| qPCR-SAT-F | 5’-CAAGATTGCCGATGCGTTGT-3’ |
| qPCR-SAT-R | 5’-GGATGCTTTCCATAGGCGGA-3’ |
| qPCR-ASO-F | 5’-TCCTCAAATCACCCAAGCCC-3’ |
| qPCR-ASO-R | 5’-GCCCTCGTAATCAGCTCCTC-3’ |
| qPCR-HL2-F | 5’-CCAGATGCTCCTGGTGGAAA-3’ |
| qPCR-HL2-R | 5’-GGCAACCATGGAAATCAGCA-3’ |
| qPCR-HL3-F | 5’-TTTGGCCGGATATGGTTGCT-3’ |
| qPCR-HL3-R | 5’-TGCCCACCTCTGTTTGTGTT-3’ |
| qPCR-HL4-F | 5’-GGTCTATGTCCATGGGGCTG-3’ |
| qPCR-HL4-R | 5’-GAGGGCTTCCCAGCAATCTT-3’ |
| qPCR-V19H-F | 5’-CTAAAGCATTGCCCCCAGGA-3’ |
| qPCR-V19H-R | 5’-GATGATGCTACGACCGCAGA-3’ |
| qPCR-MAT-F | 5’-AATCCCGACGGCCATGAAAT-3’ |
| qPCR-MAT-R | 5’-GCGGCAGTGAATTCGAACAT-3’ |
| qPCR-T19H-F | 5’-GGGGGACAGACACAACACAT-3’ |

| | |
|---|---|
| qPCR-T19H-R | 5’-AAGGATGTAACCGGAGTGCC-3’ |
| qPCR-TAT-F | 5’-TGACTTCGGATGGGGAAAGC-3’ |
| qPCR-TAT-R | 5’-GCTTCTGGTTCGGGAGAGTC-3’ |
| qPCR-TEX1-F | 5’-GGCCGGAGCCGGATATTTTT-3’ |
| qPCR-TEX1-R | 5’-AAGCTGTGCCAACGGTAGTT-3’ |
| qPCR-Redox1-F | 5’-AACATCTGCTGTTGGGGGAG-3’ |
| qPCR-Redox1-R | 5’-TAACATCCCCCTTCGCAAGC-3’ |
| qPCR-Redox2-F | 5’-GGTGTCGCTTGGGGAAGTAA-3’ |
| qPCR-Redox2-R | 5’-GAGACTTGCTCCTTGCTCGT-3’ |
| qPCR-ORCA3-F | 5’-ATACAGAAACGCGGTAGCCC-3’ |
| qPCR-ORCA3-R | 5’-CGCCCTTATACCGGTTCCAA-3’ |
| qPCR-ORCA2-F | 5’-TTGGGAACTTACGAGACGGC-3’ |
| qPCR-ORCA2-R | 5’-ACCGGAGCATTAGCAGAACC-3’ |
| qPCR-BPF-F | 5’-GCAATGAGGCCTTGTTTCCG-3’ |
| qPCR-BPF-R | 5’-TGGTCTCCTGGTCATAGGCA-3’ |
| qPCR-MYC2-F | 5’-ATCTATGCCTGCCCAACCTG-3’ |
| qPCR-MYC2-R | 5’-GGCGAATTCGACAACAGACG-3’ |

| | |
|---|---|
| qPCR-WRKY1-F | 5’-GGCTTGGCATGATCGGAAGA-3’ |
| qPCR-WRKY1-R | 5’-TTGTACGGAGAGTTCGGCAG-3’ |
| qPCR-ZCT1-F | 5’-GGCGGCAGATCTACTCCATC-3’ |
| qPCR-ZCT1-R | 5’-GGCGTCTCATATGACCTCCG-3’ |
| qPCR-ZCT2-F | 5’-TCACCGAGCGAGTCATAAGC-3’ |
| qPCR-ZCT2-R | 5’-AATTACCGGACGCCGAATCA-3’ |
| qPCR-GBF1-F | 5’-GGAAACAGGCTGAGACGGAA-3’ |
| qPCR-GBF1-R | 5’-ATCTCCCCTGCTTGCATGAC-3’ |
| qPCR-GBF2-F | 5’-CTGCTAATGGTGCTTGCTCG-3’ |
| qPCR-GBF2-R | 5’-CTGAGAACCATGAGCCCCAC-3’ |
| qPCR-GBF3-F | 5’-GTAGCTATGCCTGCGACCAA-3’ |
| qPCR-GBF3-R | 5’-GGAGCTACAGCAGATGGGAC-3’ |
| qPCR-BIS1-F | 5’-AGGATTTCGCTGCACTTGGA-3’ |
| qPCR-BIS1-R | 5’-AGTTCCGTAGCCAATGCACA-3’ |
| qPCR-BIS2-F | 5’-TGGAAGCTTGGCTCTTGACA-3’ |
| qPCR-BIS2-R | 5’-GTCTGCCATTGGTGGACTGT-3’ |
