# Supplementary figures and images for: Co-Overexpression of Geissoschizine Synthase and Catharanthine Synthase Increases Catharanthine Biosynthesis in Catharanthus roseus Hairy Roots
Source: Plants (Basel). 2026 Jul 21;15(14):2220. doi: 10.3390/plants15142220 (PMC13416060; doi:10.3390/plants15142220)

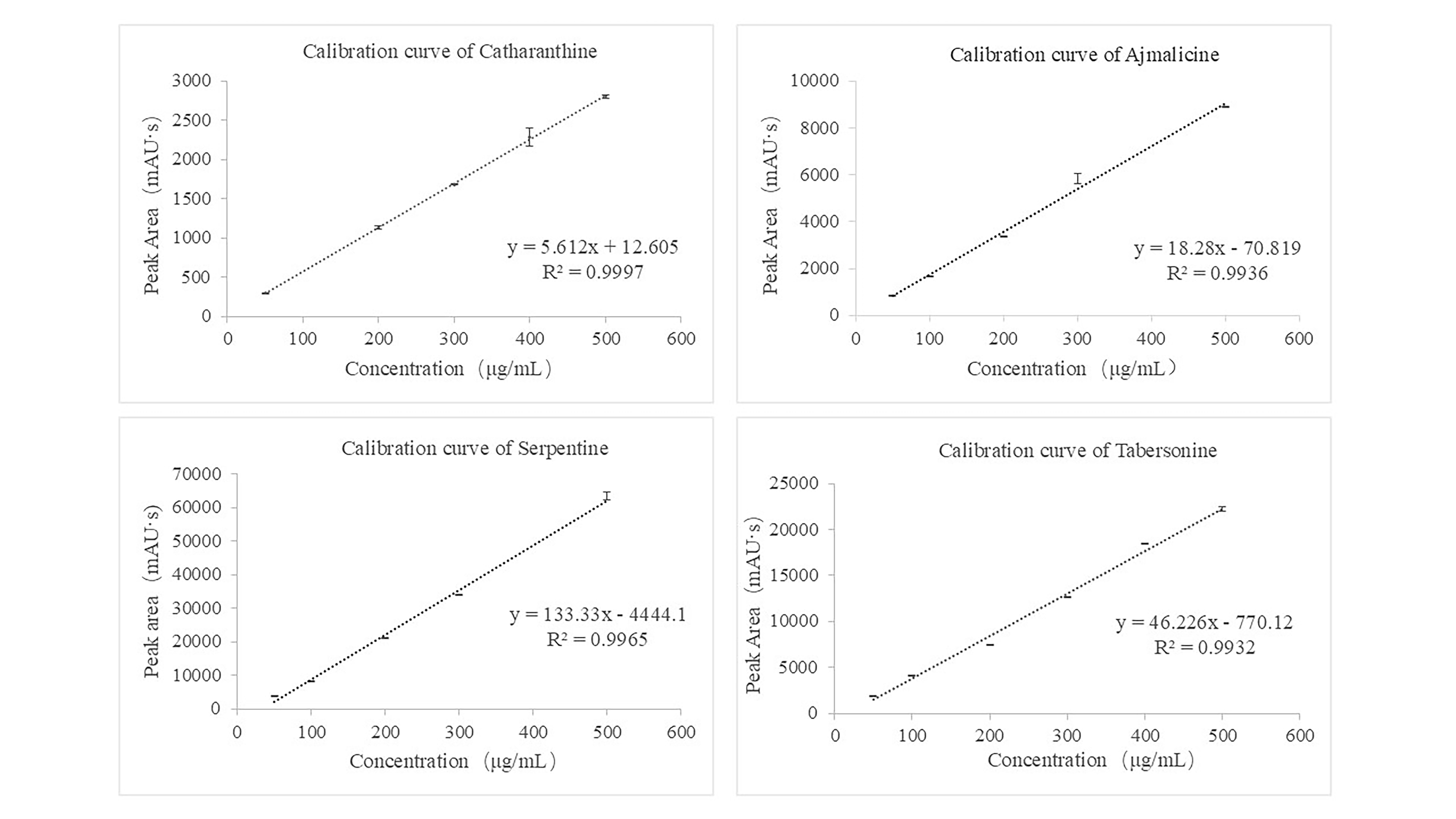

Supplement: Supplementary file 1 [file plants-15-02220-s001.zip › Supplemental Figure S1.jpeg]

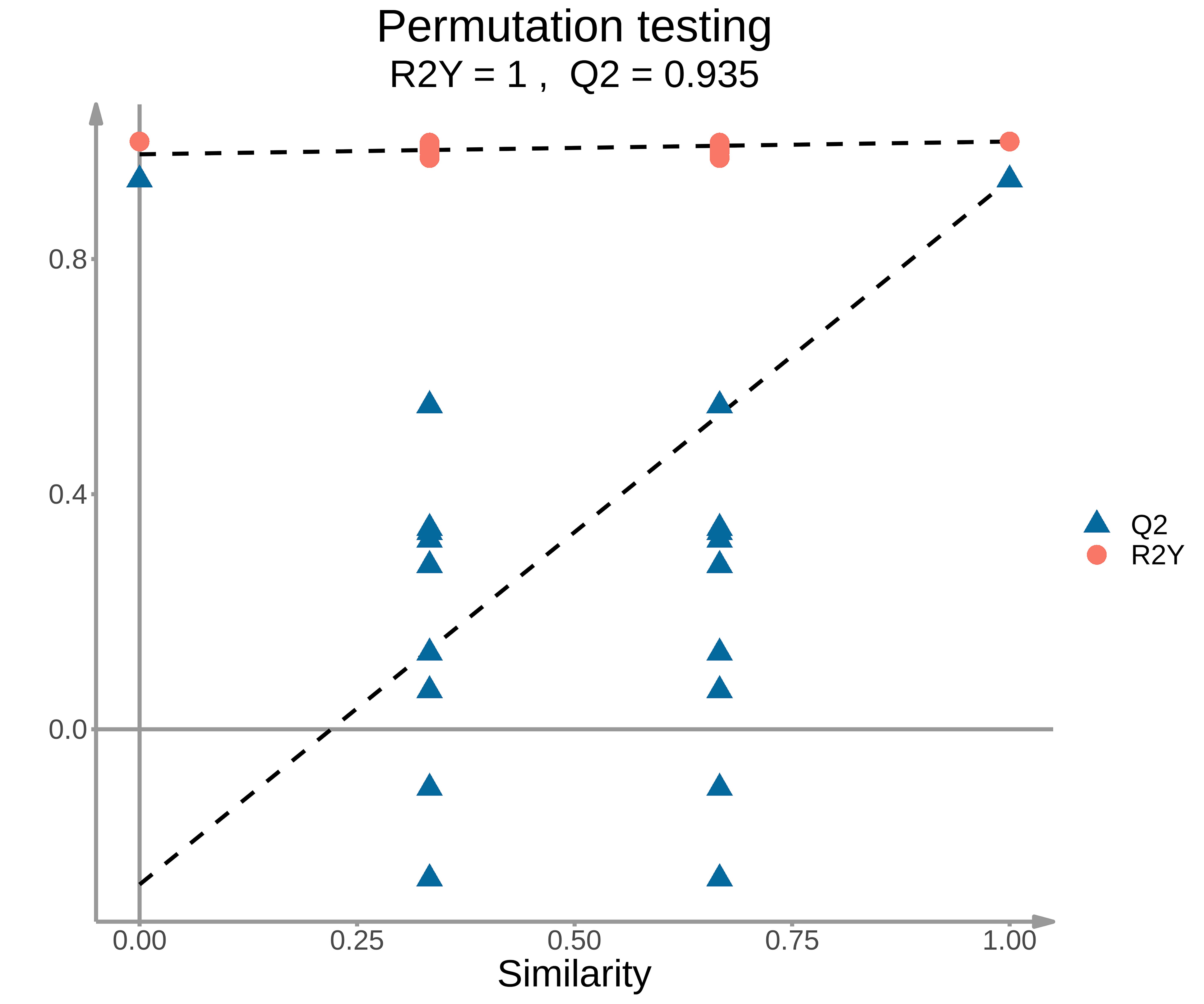

Supplement: Supplementary file 1 [file plants-15-02220-s001.zip › Supplemental Figure S2b.jpeg]

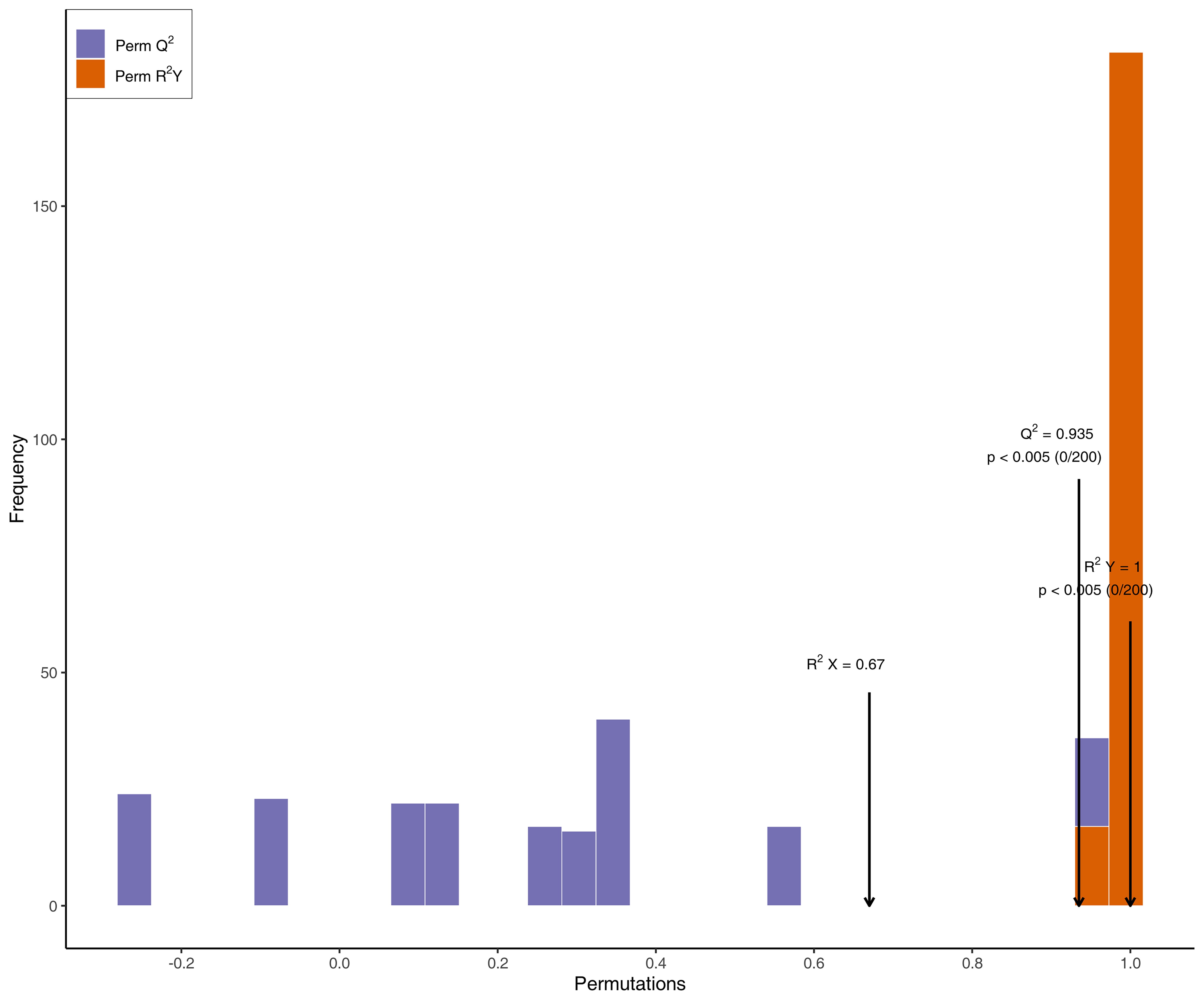

Supplement: Supplementary file 1 [file plants-15-02220-s001.zip › Supplemental Figure S2a.jpeg]
